# Fasting Blood Glucose Profile among Secondary School Adolescents in Ado-Ekiti, Nigeria

**DOI:** 10.1155/2015/417859

**Published:** 2015-03-23

**Authors:** I. O. Oluwayemi, S. J. Brink, E. E. Oyenusi, O. A. Oduwole, M. A. Oluwayemi

**Affiliations:** ^1^Department of Paediatrics, College of Medicine, Ekiti State University, Ado-Ekiti, Ekiti State, Nigeria; ^2^New England Diabetes and Endocrine Center, USA; ^3^Paediatric Endocrinology Training Centre for West Africa, Lagos University Teaching Hospital, Idi-Araba, Lagos, Nigeria; ^4^Clinical Nursing Services, Ekiti State University Teaching Hospital, Ado-Ekiti, Ekiti State, Nigeria

## Abstract

*Background.* Over the past two decades there has been an increase in type 2 diabetes mellitus (T2DM) in children. Baseline data is needed to assess the impact of changing lifestyles on Ado-Ekiti, a previously semiurban community in Southwest Nigeria. This study was therefore conducted to assess the fasting blood glucose (FBG) of adolescents in Ado-Ekiti, Nigeria. *Methodology.* This was a cross-sectional study involving 628 adolescents from three different secondary schools in Ado-Ekiti, Nigeria. With parental consent, volunteers completed a structured questionnaire, and an overnight FBG was measured. *Results.* There were 346 males and 282 females (male : female ratio = 1.2 : 1). Their ages ranged from 10 to 19 years (mean age: 14.2 ± 1.7 years). Four hundred and forty-four (70.7%) had normal FBG, while 180 (28.7%) and 4 (0.6%) had FBG in the prediabetic and diabetic range, respectively. Female gender, age group 10–14 years, and family history of obesity were significantly associated with impaired FBG (*P* value <0.001, <0.001, and 0.045, resp.). *Conclusion.* Impaired FBG is common among secondary school adolescents and it is more prevalent among younger female adolescents (10–14 years) with positive family history of obesity.

## 1. Introduction

There is rising incidence of noncommunicable diseases like diabetes, hypertension, and coronary heart diseases globally [1–3]. Increased risk of impaired glucose tolerance, insulin resistance, and type 2 diabetes (T2DM) has been found to be associated with obesity in adolescents [4]. The American Academy of Paediatrics and the American Diabetes Association have recommended that children aged 10 years or at the onset of puberty who are overweight and have at least two other risk factors should be tested every two years for T2DM [5, 6]. The risk factors for developing T2DM include family history of T2DM in first- or second-degree relative, belonging to certain ethnic groups (i.e., Native American, African American, Hispanic, Japanese, or other Asian/Pacific Islanders), or having signs associated with insulin resistance (hypertension, dyslipidemia, acanthosis nigricans, or polycystic ovarian syndrome) [5, 7]. Similarly other endogenous populations, for instance, in Canada, Australia, Russia, and Latin America, may share similar genetic predispositions [8].

Beck-Nielsen and Groop [9] proposed a three-stage model for the development of T2DM. Stage 1 includes fasting hyperinsulinemia with normal or slightly increased blood glucose, especially mild fasting hyperglycemia. Stage 2 is characterized by prediabetic glucose intolerance with insulin resistance, and Stage 3 is development of classical symptomatic or nonsymptomatic T2DM with more persistent hyperglycemia present. Many of the macrovascular changes associated with diabetes and related to cardiovascular disease (CVD) begin in Stages 1 and 2, well before overt diagnosis [10].

Adolescents are a dependent population and development of diabetes mellitus or other noncommunicable diseases will pose a burden to parents and society at large,, hence the need to continually assess the FBG of adolescents in rapidly changing communities. This will help in early detection and hopefully the control of the prediabetic phase through education and lifestyle modification. It will also help in prompt commencement of treatment in those with established diabetes to improve quality of life and prevent complications. Data on FBG among Nigerian adolescents are scarce, hence the need for the present study especially in Ado-Ekiti, a relatively new and rapidly developing state capital in Nigeria with changes in lifestyle associated with urbanization such as inappropriate dietary practices (fast food consumption, low fruit consumption) and low physical activity [11, 12].

## 2. Material and Methods

Secondary school adolescents of both sexes who satisfied the inclusion criteria were recruited for the study from three different schools in Ado-Ekiti, the capital of Ekiti State, Nigeria. Ethical clearance and permission to enter the schools were obtained from the Research and Ethics Committee of the Ekiti State University Teaching Hospital, Ado-Ekiti, and the state's Ministry of Education, respectively. Inclusion criteria for the study were apparently healthy secondary school adolescents aged between 10 and 19 years, with no history of diabetes. With parental consent, volunteers completed a structured questionnaire, and FBG was measured after an overnight fast. Capillary blood sample was collected for FBG measurement using Accu-Chek Active glucometer, after the thumb or index finger had been cleaned with wet (water) cotton wool. Data was entered and analyzed using SPSS 16.0 for Windows (SPSS Inc., Chicago, USA). Subjects were grouped based on their age, gender, family history of obesity and diabetes, and fasting blood glucose (FBG). Cross tabulation and tests for association with chi square (*χ* ^2^) were done and *P* values less than 0.05 were regarded as significant.

## 3. Results

**Figure 1:**
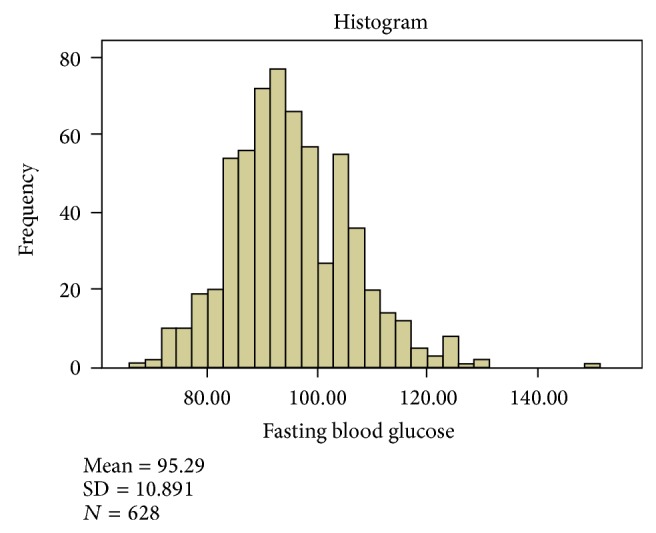
Fasting blood glucose (mg/dL) pattern in 628 secondary school adolescents.

**Figure 2:**
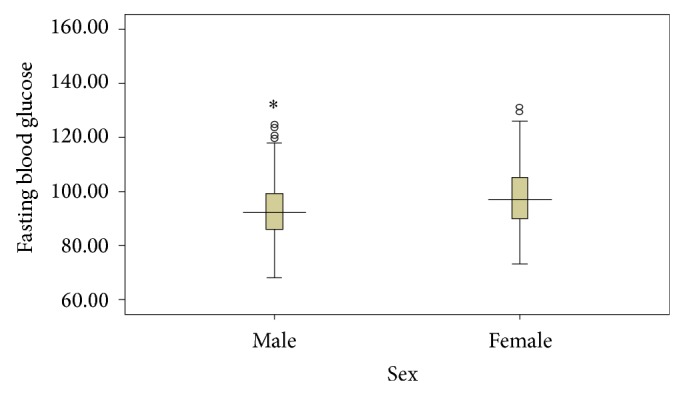
Comparison of FBG (mg/dL) pattern in male and female population.

**Figure 3:**
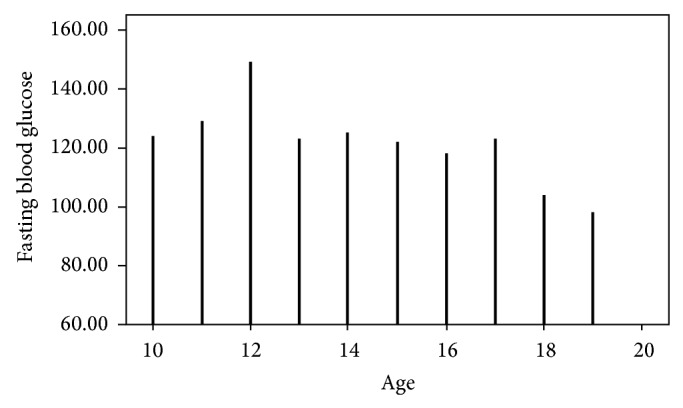
Profile of fasting blood glucose (mg/dL) according to age (years) of adolescents in Ado-Ekiti.

The subjects in the present study comprised 346 male and 282 female adolescents (male to female ratio of 1.2 : 1) with mean age of 14.2 ± 1.7 years and age range of 10 to 19 years. Four (0.6%) adolescents had FBG in diabetic range; 180 (28.7%) had impaired FBG and 121 (67%) of these were in the 10–14-year age group. Also, all the four adolescents who had diabetic FBG range were in the same 10–14-year age group (Table 1). There were 77 (41.8%) males and 107 (58.2%) females in the 184 (29.3%) adolescents with high FBG (180 in prediabetic and 4 in diabetic range) among the studied 628 adolescents giving a male to female ratio of 0.7 : 1. The proportion of males with high FBG of the overall studied male population of 346 was 77 (22.3%) while the proportion of females with high FBG of the overall female population of 282 was 107 (37.9%). This difference was statistically significant (*χ* ^2^ = 18.462, df = 1, *P* = 0.000).  Figure 1 shows the FBG profile in the studied sample of secondary school adolescents in Ado-Ekiti. The mean (SD) for their FBG was 95.3 (10.9), median was 94.0, and it ranged from 68 to 149 mg/dL. The variability of FBG in both genders was comparable except that the median FBG for females was slightly higher (Figure 2). The mean (SD) FBG for males was 93.4 (10.8) and for females was 97.6 (10.5). The FBG chart for age (Figure 3) showed two distinct peaks which occurred at 12 and 17 years though there appears to be a little rise at the age of 14 years. Female gender, early adolescence (age group 10–14 years), and family history of obesity have statistically significant influence on FBG levels (*P* value <0.001, <0.001, and 0.045, resp.).

## 4. Discussion

The review of the fasting blood glucose of the adolescents in Ado-Ekiti showed that the majority (70.7%) had normal FBG, and a rather large number, about a third (28.7%), had impaired FBG in the prediabetic range and 0.6% of them had presumed diabetic FBG range. The possibility of secondary school adolescents falsely denying taking some juice or snack before the test cannot be completely ruled out and this may contribute to the unusually high proportion of those with impaired FBG in this study. There may also be some bias using plasma meter BG readings rather than more centralized sampling systems as reported in other studies [2, 13]. Some nervousness of participants not used to finger pricks may also have contributed to some false elevations and repeat testing will be proposed to double-check these results. The FBG profile of the adolescents plotted against their ages showed two distinct peaks which occurred at 12 and 17 years (Figure 3); this is similar to the peaks in their BMI which occurred at the same ages of 12 and 17 years. This finding is in concordance with findings among adolescents in Beijing area of China [14] whose FBG peaked at 12-13 years and at 17 years of age. Increased secretion of growth hormone and adrenocortical and gonadal hormones during puberty usually causes increase in insulin resistance and this could explain the peaks in FBG of adolescents at 12 and 17 years which roughly correspond to early and late phase of puberty in both genders combined [13]. It was also demonstrated in this index study that early adolescence (age group 10–14 years), female sex, and family history of obesity have statistically significant association with impaired fasting blood glucose and this finding agrees with findings from previous studies [13, 14]. Moreover, the mean FBG in the male and female adolescents in index study (93.4 ± 10.8; 97.6 ± 10.5 mg/dL, resp.) is slightly higher than those of Moroccan [2] male and female adolescents of a similar age (92 ± 14.0; 89 ± 10.0 mg/dL, resp.). This difference may be due to different methods used for analysis of FBG. In the Moroccan study, Mehdad et al. [2] used hexokinase method to determine FBG while the current study assessed capillary FBG with Accu-Chek Active glucometer. Also, the Moroccan study [2] had a smaller and disproportionate study population (44 males; 123 females) compared to the index study of 346 male and 282 female adolescents.

*Strength and Limitation of the Study*

1. The sample size for this study is adequate and both genders were well represented.
2. The population of adolescents available for this study from private secondary school was small compared to those from public secondary schools, hence preventing meaningful comparison.

## 5. Conclusion

The present study among secondary school adolescents in Ado-Ekiti, Nigeria, showed that FBG was normal in two-thirds of them while the remaining one-third had impaired FBG. Also, impaired FBG is significantly more common among younger female adolescents (10–14 years) with positive family history of obesity.

## Figures and Tables

**Table 1:** Fasting blood glucose grading according to age group.

Age group	Fasting blood glucose (FBG) grading			Total
	Euglycemia	Impaired FBG (prediabetic)	Diabetes FBG	
	2.8–5.5 mmol/L	5.6–6.9 mmol/L	≥7 mmol/L	
	(50–99 mg/dL)	(100–125 mg/dL)	(126 mg/dL)	
10–14 years	232	121	4	357
15–19 years	212	59	0	271

Total (%)	444 (70.7)	180 (28.7)	4 (0.6)	628 (100)
